# Assessment of Antioxidant Capacity and Cytotoxicity of Selected Malaysian Plants

**DOI:** 10.3390/molecules15042139

**Published:** 2010-03-25

**Authors:** Lai Teng Ling, Ammu Kutty Radhakrishnan, Thavamanithevi Subramaniam, Hwee Ming Cheng, Uma D. Palanisamy

**Affiliations:** 1 Department of Physiology, Faculty of Medicine, University of Malaya, 50603, Kuala Lumpur, Malaysia; E-Mail: linglaiteng@gmail.com (L.T.L.); 2 Department of Pathology, Faculty of Medicine, Pharmacy and Health Sciences, International Medical University, Bukit Jalil, 57000, Kuala Lumpur, Malaysia; E-Mail: ammu_radhakrishnan@imu.edu.my (A.K.R.); 3 SIRIM Bhd, 1 Persiaran Dato Menteri, 40911, Shah Alam, Selangor Darul Ehsan, Malaysia; E-Mail: thava@sirim.my (T.S.); 4 School of Medicine and Health Sciences, Monash University Sunway Campus, Jalan Lagoon Selatan, 46150, Bandar Sunway, Malaysia

**Keywords:** free radical scavenging, lipid peroxidation, cytotoxicity, total phenolic content, elemental analysis, Malaysian plants

## Abstract

Thirteen Malaysian plants; *Artocarpus champeden, Azadirachta indica, Fragaria x ananassa, Garcinia mangostana, Lawsonia inermis, Mangifera indica, Nephelium lappaceum, Nephelium mutobile, Peltophorum pterocarpum, Psidium guajava* and *Syzygium aqueum*, selected for their use in traditional medicine, were subjected to a variety of assays. Antioxidant capability, total phenolic content, elemental composition, as well as it cytotoxity to several cell lines of the aqueous and ethanolic extracts from different parts of these selected Malaysian plants were determined. In general, the ethanolic extracts were better free radical scavengers than the aqueous extracts and some of the tested extracts were even more potent than a commercial grape seed preparation. Similar results were seen in the lipid peroxidation inhibition studies. Our findings also showed a strong correlation of antioxidant activity with the total phenolic content. These extracts when tested for its heavy metals content, were found to be below permissible value for nutraceutical application. In addition, most of the extracts were found not cytotoxic to 3T3 and 4T1 cells at concentrations as high as 100 μg/mL. We conclude that although traditionally these plants are used in the aqueous form, its commercial preparation could be achieved using ethanol since a high total phenolic content and antioxidant activity is associated with this method of preparation.

## 1. Introduction

Antioxidants are either naturally found in the body or synthetically produced to be used in the food industry. A wide range of antioxidants, both natural and synthetic have been proposed for use in the treatment of human diseases. Butylated hydroxyanisole (BHA), butylated hydroxytoluene (BHT), propyl gallate (PG) and tert-butylhydroquinone (TBHQ) are the synthetic antioxidants added to food to stabilize them and prevent off-flavor development. However, the use of these synthetic antioxidants is restricted in some countries due to their toxicity [1]. Consequently there is a need to explore new natural sources of antioxidants to replace synthetic antioxidants.

Plants that contain active components, namely phenolics and polyphenolics, have raised public interest in their potential to act as antioxidants. Phenolic compounds constitute large, heterogeneous groups of secondary plant metabolites derived from phenylalanine or tyrosine and extensively distributed in the plant kingdom [2]. There are numerous studies that prove the positive relationship of consumption of fruits and vegetables containing phenolics to the prevention of aging related diseases caused by oxidative stress [3,4].

Phenolics in green tea, fruits and vegetables, grape seeds and skin have been recognized as natural antioxidants and have been extensively studied by many investigators for their noteworthy role as antimicrobial agents, for their antiallergenic and anti-inflammatory properties along with their antimutagenic action [5,6].

Due to these numerous advantages for humans, it has become a major concern to establish the levels of some constituents and heavy metals in common herbal plants. The presence of heavy metals at high levels can be detrimental to human health [7]. The World Health Organization [8] has also emphasized the need to ensure the quality control of plant products by using modern techniques. Thus the analytical control of selected heavy trace metals and elemental analysis of plants, especially medicinal plants, is part of the quality control, to determine their purity, safety and efficacy.

In addition, studies have shown that some plant extracts contain certain compounds that are potentially toxic to humans and animals. Some of the reported cytotoxic compounds include alkaloids, glycosides, oxalates, phytotoxins (toxalbumins), resins, essential oils, amino acids, furanocoumarins, polyacetylenes, protein, peptides, coumarins, flavonoids and glycosides [9]. Therefore, evaluating its cytotoxicty is important to assess a plant’s suitability for its potential commercial applications, particularly as a nutraceutical.

To date, a huge number of plants have been studied as potential sources of antioxidants. However, there are no reports on the antioxidant activity, heavy metal and elemental analysis and cytotoxicity of Malaysian plants which are potential sources of new antioxidants. The objective of this study was to evaluate selected Malaysian plants for their free radical scavenging and inhibition in lipid peroxidation activity, and phenolic content. This was followed by a heavy metal and elements analysis and an examination of their cytotoxicity against 4T1 and 3T3 cell lines. The plants were chosen based on their use for traditional applications within the region. The investigation is essential to establish the phenolic content of selected Malaysian plants, their capability as potential antioxidants and to ensure their safe use.

## 2. Results and Discussion

### 2.1. Total phenolic content

The plants were initially characterized by the amount of phenolic compound (expressed as mg gallic acid/1 g plant) they contained. All ethanolic extracts showed a higher total phenolic content compared to the corresponding aqueous counterparts (Table 1). This corroborates the findings in the literature [10]. The total phenolic content of a few of the extracts from the various species were higher than the commercial preparation of grape seed (*Peltophorum pterocarpum* bark > *Nephelium lappaceum* peel > *Mangifera indica* leaf > grape seed).

### 2.2. Free radical scavenging activity

Amongst the three different free radical scavenging (FRS) assays used in this study to assess ethanolic extracts (Table 1), the ABTS was seen to be the most sensitive assay followed by the Galvinoxyl one and finally the DPPH scavenging assay. This was because in most of the extracts the ABTS assay showed the highest 1/IC_50_ values. On the other hand both the ABTS and Galvinoxyl analyses were just as sensitive in the case of the aqueous extracts. It should be noted that the DPPH scavenging assay is the most commonly used one due to its ease of execution and high reproducibility.

Similar results were observed by Buenger and colleagues [11] who showed that DPPH assay is easy to implement and yielded reproducible results. The activity rankings of the ethanolic plant extracts from the different assay methods showed a strikingly similar trend with *Garcinia mangostana*, *Nephelium lappaceum* (peel), *Mangifera indica* and *Psidium guajava* extracts exhibiting the highest antioxidant activity. Aqueous extracts on the other hand, did not show superior antioxidant activity (Table 2). This could be due to the higher phenolic content in the ethanolic extracts as compared to the aqueous extracts. It was also encouraging to note that most of the plant extracts studied exhibited higher 1/IC_50_ values compared to the commercial grape seed extract.

**Figure 1:**
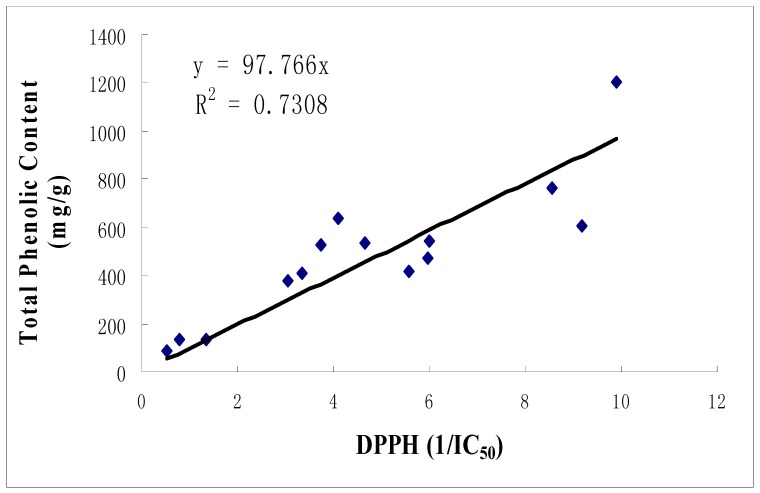
Correlation between DPPH scavenging activity and total phenolic content.

The correlation between total phenolic content and DPPH scavenging activity for ethanolic extracts was observed to be high, with a correlation, R^2^=0.7308 (Figure 1) indicating that phenolic compounds may be the major contributor of the scavenging activity of the extracts. This has been shown in other studies where antioxidant capacity of plant extracts correlated well with the total phenolic content [12,13,14].

**Table 2:** Free radical scavenging activity of various Malaysian plants extracts in ethanol.

Ethanolic extracts	Plant Part	Assays (1/IC_50_, mg/mL)		
		DPPH	Galvinoxyl	ABTS
*Azadirachta indica*	leaf	1.35± 0.46	4.85 ± 0.08	6.01 ± 0.04
*Mangifera indica*	leaf	5.99 ± 0.02	19.86 ± 0.01	46.56 ± 0
*Garcinia mangostana*	peel	9.19 ± 0.02	37.56 ± 0.01	41.85 ± 0.02
*Nephelium lappaceum*	peel	8.56 ± 0.05	85.41 ± 0.01	62.99 ± 0
*Psidium guajava*	leaf	5.56 ± 0.08	30.88 ± 0.02	46.76 ± 0
*Fragaria x ananassa*	leaf	0.54 ± 0.80	2.53 ± 0.21	3.24 ± 0.11
*Lawsonia inermis*	leaf	0.79 ± 0.18	3.1 ± 0.23	8.83 ± 0.04
*Syzygium aqueum*	leaf	4.65 ± 0.02	11.98 ± 0.01	34.52 ± 0
*Nephelium lappaceum*	leaf	3.04 ± 0.03	71.04 ± 0.03	8.3 ± 0.07
*Peltophorum pterocarpum*	leaf	5.96 ± 0.12	34.76 ± 0.02	11.53 ± 0.08
*Peltophorum pterocarpum*	bark	9.90 ± 0.04	33.52 ± 0.01	9.11 ± 0.09
*Artocarpus champeden*	leaf	3.34 ± 0.21	11.54 ± 0.1	12.39 ± 0.04
*Nephelium mutobile*	leaf	4.10 ± 0.03	18.5 ± 0.04	10 ± 0.04
Grape seed (commercial source)	seed	3.75 ± 0.10	10.54 ± 0.03	28.18 ± 0.01

Values are the mean ± standard deviation (n = 3).

**Table 3:** Free radical scavenging activity of various Malaysian plants extracted in water.

Aqueous Extracts	Plant Part	Assays (1/IC_50_, mg/mL)		
		DPPH	Galvinoxyl	ABTS
*Azadirachta indica*	leaf	1.05 ± 0.14	2.96 ± 0.1	2.41 ± 0.23
*Mangifera indica*	leaf	2.02 ± 0.39	4.65 ± 0.01	8.21 ± 0.03
*Garcinia mangostana*	peel	0.6 ± 2.36	1.39 ± 1.18	1.8 ± 0.76
*Nephelium lappaceum*	peel	1.86 ± 0.15	6.81 ± 0.06	5.46 ± 0.06
*Psidium guajava*	leaf	4.56 ± 0.01	7.5 ± 0.09	5.4 ± 0.03
*Fragaria x ananassa*	leaf	2.69 ± 0.07	3.56 ± 0.2	5.3 ± 0.11
*Lawsonia inermis*	leaf	0.27 ± 0.34	0.83 ± 0.32	1.08 ± 0.07
*Syzygium aqueum*	leaf	3.07 ± 0.07	6.68 ± 0.04	5.22 ± 0.07
*Nephelium lappaceum*	leaf	1.49 ± 0.02	3.64 ± 0.09	3.96 ± 0.06
*Peltophorum pterocarpum*	leaf	6.21 ± 0.05	30.85 ± 0.01	9.63 ± 0.02
*Peltophorum pterocarpum*	bark	5.05 ± 0.12	24.58 ± 0.01	9.02 ± 0.04
*Artocarpus champeden*	leaf	4.54 ± 0.01	14.33 ± 0.04	9.52 ± 0.03
*Nephelium mutobile*	leaf	0.27 ± 0.27	1.25 ± 0.09	0.9 ± 0.37
Grape seed (commercial source)	seed	2.18 ± 0.18	2.07 ± 0.33	5.19 ± 0.12
Vitamin C	NA	34.45 ± 0.04	NA	NA
Emblica ^TM^	NA	3.36 ± 0.05	NA	NA

Values are the mean± standard deviation (n = 3).

### 2.3. Inhibition of Lipid Peroxidation

The ability of the various species to inhibit lipid peroxidation was once again seen to be higher in the ethanolic extracts. The highest activity seen in grape seed ethanolic extract with a 1/IC_50_ of 16.76 ± 0.02mg/ml (Table 4) and has been proved by previous animal study that treatment with grape seed extract suppresses lipid peroxidation and reduces hypoxic ischemic brain injury in neonatal rat [15]. The Malaysian plant extracts studied did not show a comparable inhibition of lipid peroxidation of grape seed extract. The highest activity being in the *Garcinia mangostana* peel > *Peltophorum pterocarpum* leaf > *Nephelium lappaceum* peel > *Nephelium mutobile* leaf > *Mangifera indica* leaf.

### 2.4. Elemental Analysis

It is important to ensure that plants that are consumed in traditional practice do not contain heavy metals or are at permissible levels. Table 3 shows the elemental content of the powderized plants. The results clearly show that almost all the plants studied contained a much lower lead (Pb), Arsenic (As) and Mercury (Hg) content compared to the permissible levels as indicated in the Food Act 1983 (ACT 281) & Regulations [16] and European regulation on food supplements [17]. Several other plants also have some lead content, however lower than permissible levels which could be due to the trees being contaminated with lead from fuel. It should be noted that *Peltophorum pterocarpum* bark and *Azadirachta indica* leaf showed a significant amount of arsenic and mercury content respectively. The results indicate that it is important to monitor the heavy metal content to ensure the safety of plant parts, particularly those that are used in traditional application.

*Mangifera indica* leaf displayed high Calcium (40937mg/kg) content compared with other extracts while *Fragaria x ananassa* leaf showed high Potassium (29805mg/kg) and Magnesium content (3532mg/kg). It is worthy to note that the *Peltophorum pterocarpum* showed a significant content of Iron (1602mg/kg) particularly in its leaf.

**Table 5:** Elemental analysis of selected Malaysian plant extracts.

Extracts	Plant Parts	Type of Tests (mg/kg)							
		Lead (Pb)	Arsenic (As)	Mercury (Hg)	Calcium (Ca)	Iron (Fe)	Potassium (K)	Magnesium (Mg)	Sodium (Na)
Malaysian Standards*	NA	10	5	0.5	NA	NA	NA	NA	NA
European Standards^**^	NA	3	1	0.1	NA	NA	NA	NA	NA
*Azadirachta indica*	leaf	0.52 ± 0.02	<0.01	0.22 ± 0.02	29217 ± 536	12 ± 3	14883 ± 253	2332 ± 146	<852
*Mangifera indica*	leaf	< 0.15	<0.01	< 0.02	40937±423	50 ± 5	4975 ± 123	2693 ±125	<905
*Garcinia mangostana*	peel	0.62 ± 0.03	<0.01	<0.02	1130 ± 53	<1.5	7862 ± 65	495 ± 25	<902
*Nephelium lappaceum*	peel	0.36 ± 0.02	<0.01	< 0.02	5073 ± 83	<1.4	5510 ± 63	1402 ± 75	<847
*Psidium guajava*	leaf	1.2 ± 0.1	<0.01	< 0.02	20038 ± 536	55 ± 5	6022 ± 763	2508 ± 128	<875
*Fragaria x ananassa*	leaf	0.27 ± 0.02	<0.01	< 0.02	15540 ± 866	<1.5	29805 ± 986	3532 ± 253	<901
*Lawsonia inermis*	leaf	0.64 ± 0.03	<0.01	< 0.02	13276 ± 654	17 ± 3	6214 ± 532	3003 ± 531	<887
*Syzygium aqueum*	leaf	0.21 ± 0.02	<0.01	< 0.02	9649 ± 423	<1.5	8223 ± 332	2347 ± 75	<908
*Nephelium lappaceum*	leaf	<0.15	<0.01	< 0.02	27507 ± 1536	6.8 ± 0.1	6165 ± 56	2597 ± 23	<891
*Peltophorum pterocarpum*	leaf	<0.5	1.95 ± 0.1	0.02 ± 0.01	3383 ± 353	1602±21	7564 ± 86	3363 ± 47	<913
*Peltophorum pterocarpum*	bark	<0.5	3.86 ± 0.2	<0.01	15110 ± 875	196 ± 10	1670 ± 63	1123 ± 46	<857
*Artocarpus champeden*	leaf	<0.5	0.4 ± 0.03	<0.01	1580 ± 632	<1.5	7601 ± 96	2521 ± 53	<910
*Nephelium mutobile*	leaf	<0.5	0.57 ± 0.03	0.03 ± 0.01	3748 ± 84	<1.4	4722 ± 86	2390 ±63	<866

* In accordance to Food Act 1983 (ACT 281) specifications and regulations; ** In accordance to European Regulation on food supplements; Values are the mean± standard deviation (n = 3).

### 2.5. Cytotoxicity activity of the plant extracts

**Figure 2:**
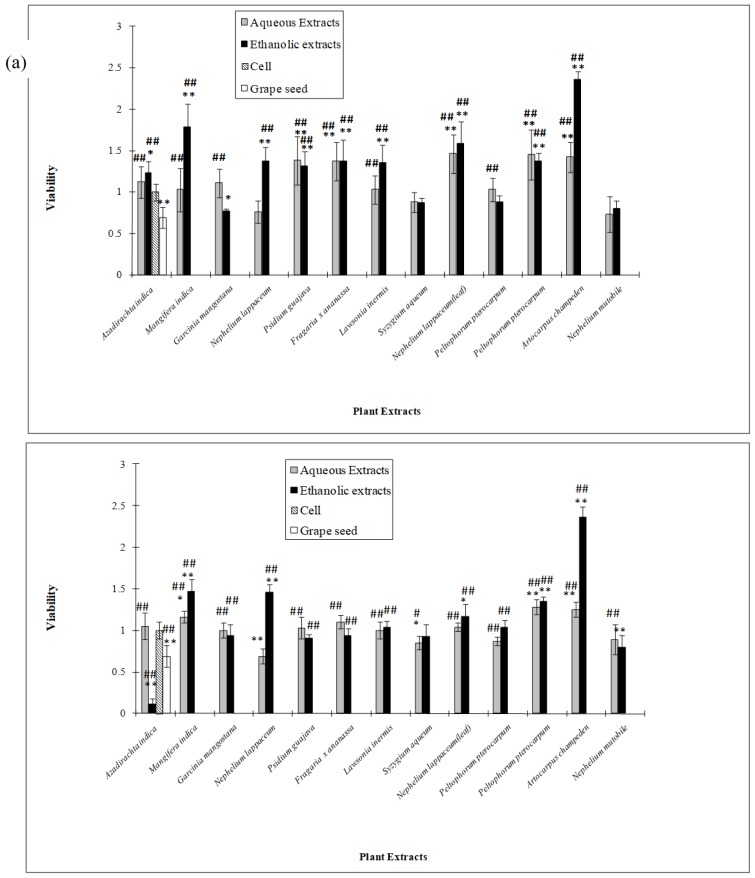
Cytotoxicity activity of selected Malaysian plants on cells at concentration of 100 µg/mL: (a) On 3T3 cells (b) on 4T1 cells.

In the cytotoxicity studies, 3T3 (mouse embryonic fibroblasts cell) and 4T1 (mouse breast cancer cell) were used. 3T3 cell is widely used to study the cytotoxicity effect of compounds and plants in *in vitro* models [18,19]. 4T1 cell is well established in our laboratory to evaluate the antiproliferative effect of extracts as a potential anticancer agent. Most of the ethanolic and aqueous extracts did not exhibit any anti-proliferative effects on 4T1 and 3T3 cells at both 50 µg/mL (data not shown) and 100 µg/mL, as shown in Figure 2a and 2b. However, *Azadirachta indica* leaf ethanolic extract showed significant inhibition of cell proliferation of 4T1 cells. The results indicate that the extract may have anti-cancer properties (Figure 2a) and was shown in previous studies where *Azadirachta indica* leaf preparation was found to activate natural killer (NK) cells (CD56^+^CD3^−^) to enhance their cytotoxic ability to tumor cells and stimulate the release of interleukin-12 (IL-12) from macrophages from healthy individuals and head-and-neck squamous cell carcionoma patients [20]. Almost all the ethanolic and aqueous extracts were not toxic to the cells at 100 µg/mL. In fact, ethanolic and aqueous extracts of *Artocarpus champeden* were seen to promote cell proliferation.

## 3. Experimental

### General

Fresh Malaysian plants were obtained from Klang Valley in Malaysia. The plants used were as follows with their common names in brackets: *Artocarpus champeden* (chempedak), *Azadirachta indica* (neem), *Fragaria x ananass* (strawberry), *Garcinia mangostana* (mangosteen), *Lawsonia inermis* (henna), *Mangifera indica* (mango), *Nephelium lappaceum* (rambutan), *Nephelium mutobile* (pulasan), *Peltophorum pterocarpum* (yellow flamboyant), *Psidium guajava* (guava) *and Syzygium aqueum* (water apple). The plants were authenticated by a botanist at the Herbarium of the Forest Research Institute of Malaysia (FRIM) in Kepong, Malaysia. The plants were extracted as reported previously [21].

Antioxidant assays using DPPH (1,1-diphenyl-2-picrylhydrazyl), Galvinoxyl, and ABTS (2,2-azino-bis-3-ethylbenzothiazoline 6-sulfonate) free radicals and lipid peroxidation were assessed according to the modified method reported previously [21]. Total phenolic content was determined using the Folin-Ciocalteu method [22], which is based on a colorimetric oxidation and reduction reaction. One mL aliquots of the extract at defined concentrations (0.01 - 5mg/mL) were added to 5 mL of Folin-Ciocalteu reagent. After 3 minutes, 4 mL of 7.5% Na_2_CO_3_ solution was added to the mixture and thoroughly mixed. The absorbance at 765 nm was taken after one hour. The blank consisted of Folin-Ciocalteu reagent (5 mL), ethanol/distilled water (1 mL) and 7.5% Na_2_CO_3_ solution (4 mL). A linear dose response regression was generated using absorbance reading of gallic acid at the wavelength of 765 nm. The calibration curve using gallic acid was obtained in the same manner as above except that the absorbance was read after 30 minutes. Total phenolic content of the extracts was calculated and the content of phenolic compounds in a respective sample was expressed in mg/g of extract, gallic acid equivalent (GAE).

Powdered samples (0.2 g) were digested using a combination of HNO_3_, HCl, and HF using the microwave digestion system as per EPA Method SW846 – 3052 [23]. After digestion was completed, the acids were evaporated and samples were brought up to 50 mL volume in 2% HNO_3_. Samples were analyzed on a PerkinElmer Optima 3000XL ICP-OES for lead, arsenic, calcium, iron, potassium, magnesium and sodium via EPA Method SW846 – 6010B [24]. The cold vapor atomic absorption spectrometer (CV-AAS) method was employed for Hg analysis after sample digestion in acid solution analyzed using EPA Method 245.6 [25].

Cytotoxicity activity of selected Malaysian plant extracts at 50 µg/mL and 100µg/mL were tested by measuring the cell proliferation of 3T3 and 4T1 cells cultured in 96-well culture plates in the presence and absence of the extracts for 24 hours at 37 °C in a humidified 5% CO_2_ incubator as described in Palanisamy and colleagues [26]. Results were expressed as mean ± standard error. Statistical comparisons between groups were performed with Student’s t-test for independent observations. Differences were considered significant at p < 0.05.

## 4. Conclusions

The plant extracts studied are a potential source of natural antioxidants. Generally, ethanolic extracts showed better activity in FRS and inhibition of lipid peroxidation assays compared to their corresponding aqueous extracts. In addition, the FRS activity of the extracts was higher than that of a commercial grape seed preparation. Most of the selected plant extracts showed hardly any heavy metal contamination in the powdered plants, and some extracts even showed the presence of essential trace minerals. The majority of the plant extracts did not exhibit anti-proliferative effects on cultured mouse fibroblast and breast cancer cells indicating that most of the plant extracts are not cytotoxic to the cells studied. We also observed a positive correlation between the ethanolic extract, phenolic content and antioxidant activity. Ethanolic extraction of plant bioactives displayed a higher yield compared to the aqueous extract [27]. Similar results were seen in our previous finding [26], where increased extraction yields were observed in ethanolic extracts and correlated strongly to its antioxidant activity and total phenolic content. Silva Pinto and co-workers [28] recently showed that ethanolic extracts of *Gingko bilibo* L. leaves displayed higher α-glucosidase and α-amylase inhibition activity than its aqueous extracts. We can therefore conclude that although traditionally these plants are used in the aqueous form, their commercial preparation could be achieved using ethanol since a high total phenolic content and antioxidant activity is associated with this method of preparation. It is desirable that these extracts be further purified to gain a better understanding of the active compounds contributing to their antioxidant activity.

## Figures and Tables

* designates a significant difference from cell alone (P < 0.05),* * designates a significant difference from cell alone (P < 0.01), # designates a significant difference from grape seed (P<0.05), ## designates a significant difference from grape seed (P < 0.01).

**Table 1:** Total Phenolic content of selected Malaysian plants.

Plant	Plant Part	Phenolic content (mg/g GA equivalent)	
		Aqueous Extract	Ethanolic Extract
*Azadirachta indica*	leaf	112 ± 13	139 ± 64
*Mangifera indica*	leaf	115 ± 14	648 ± 106
*Garcinia mangostana*	peel	135 ± 96	249 ± 135
*Nephelium lappaceum*	peel	212 ± 68	762 ± 60
*Psidium guajava*	leaf	211 ± 38	421 ± 114
*Fragaria x ananassa*	leaf	79 ± 15	86 ± 21
*Lawsonia inermis*	leaf	55 ± 17	135 ± 28
*Syzygium aqueum*	leaf	186 ± 44	524 ± 176
*Nephelium lappaceum*	leaf	194 ± 19	380 ± 40
*Peltophorum pterocarpum*	leaf	134 ± 22	475 ± 99
*Peltophorum pterocarpum*	bark	272 ± 117	1204 ± 602
*Artocarpus champeden*	leaf	279 ± 76	410 ± 128
*Nephelium mutobile*	leaf	53 ± 33	127 ± 3
Grape seed (commercial source)	seed	130 ± 7	605 ± 33

**Table 4:** Inhibition of lipid peroxidation of various extracted Malaysian plants in water and ethanol.

Plants	Plant Part	Assay (1/IC_50_, mg/mL)	
		Aqueous Extract	Ethanolic Extract
*Azadirachta indica*	leaf	0.44 ± 2.38	1.4 ± 0.34
*Mangifera indica*	leaf	0.72 ± 0.95	3.11 ± 0.13
*Garcinia mangostana*	peel	3.88 ± 0.01	5.86 ± 0.05
*Nephelium lappaceum*	peel	1.44 ± 0.09	3.42 ± 0.1
*Psidium guajava*	leaf	2.56 ± 0.05	1.61 ± 0.18
*Fragaria x ananassa*	leaf	0.35 ± 1.86	0.32 ± 0.95
*Lawsonia inermis*	leaf	0.2 ± 0.2	0.3 ± 0.13
*Syzygium aqueum*	leaf	1.2 ± 0.09	2.4 ± 0.06
*Nephelium lappaceum*	leaf	0.69 ± 0.49	2.64 ± 0.1
*Peltophorum pterocarpum*	leaf	1.29 ± 0.15	2.74 ± 0.07
*Peltophorum pterocarpum*	bark	3.65 ± 0.1	5.68 ± 0.06
*Artocarpus champeden*	leaf	3.73 ± 0.05	2.08 ± 0.24
*Nephelium mutobile*	leaf	0.32 ± 1.19	3.35 ± 0.08
Grape seed (commercial source)	seed	1.99 ± 0.19	16.76 ± 0.02

Values are the mean ± standard deviation (n = 3).

## References

[1] Huang H.L., Wang B.G. (2004). Antioxidant capacity and lipophilic content of seaweeds collected from the qingdao coastline. J. Agric. Food Chem..

[2] Shahidi F., Naczk M. (2006). Food Phenolics: Phenolics in cereal, fruits and vegetables: Occurrence, extraction and analysis. J. Pharm. Biomed. Anal..

[3] Kaur C., Kapoor H.C. (2001). Antioxidants in fruits and vegetables-the millennium’s health. Int. J. Food Sci. Technol..

[4] Vinson A., Xuehui S., Ligia Z., Bose P. (2001). Phenol antioxidant quantity and quality in foods: Fruits. J. Agric. Food Chem.

[5] Rice-Evans C.A., Miller N.J., Paganga G. (1996). Structure-antioxidant activity relationships of flavonoids and phenolic acids. Free Radic. Biol. Med..

[6] Mukai K., Nagai S., Ohara K. (2005). Kinetic study of the quenching reaction of singlet oxygen by tea catechins in ethanol solution. Free Radic. Biol. Med..

[7] Schumacher M., Bosque M.A., Domingo J.L., Corbella J. (1991). Dietary intake of lead and cadmium from foods in Tarragona Province, Spain. Bull. Environ. Contam. Toxicol..

[8] WHO (1992). Expert committee on specification for pharmaceuticals preparation.

[9] Orech F.O., Akenga T., Ochora J., Friis H., Aagaard-Hansen J. (2005). Potential toxicity of some traditional leafy vegeteables consumed in Nyang’Oma division, western kenya. Afr. J. Food Agric. Nutr. Dev..

[10] Jacopic J., Veberic R., Stampar F. (2009). Extraction of phenolic compounds from green walnut fruits in different solvents. Acta Agric. Slov..

[11] Buenger J., Ackermann H., H-Jentzsch A., Mehling A., Pfitzner I., Reiffen K.A., Schroeder K.R., Wollenweber U. (2006). An interlaboratory comparison of methods used to assess antioxidant potentials. Int. J. Cosmet. Sci..

[12] Gao X., Bjork L., Trajkovski V., Uggla M. (2000). Evaluation f antioxidant activities of rosehip ethanol extracts in different test systems. J. Sci. Food Agric..

[13] Li H.B., Wong C.C., Cheng K.W., Chen F. (2008). Antioxidant properties *in vitro* and total phenolic contents in methanol extracts from medicinal plants. LWT Food Sci. Technol..

[14] Jayaprakasha G.K., Jaganmohan R.L. (2000). Phenolic constituents from the lichen parmotrema stuppeum (Nyl.) Hale and their antioxidant activity. Z. Naturforsch., C, J. Biosci..

[15] Feng Y.Z., Liu Y.M., Fratkins J.Dm., LeBlanc M.H. (2005). Grape seed extract suppresses lipid peroxidation and reduces hypoxic ischemic brain injury in neonatal rats. Brain Res. Bull..

[16] (2004). Food Act 1983 (ACT 281) & Regulations.

[17] EC (2006). Regulation No.1881/2006. Setting Maximum Levels for Certain Contaminants in Food Stuffs.

[18] Melt M.L., Waugh J., Schneider S., Greene N.D., Rodriguez C., Hare C. (2006). Mammalian cell cytotoxicity of diesel engine emission fractions. J. Appl. Toxicol..

[19] Rucinska A., Roszczyk M., Gabryelak T. (2008). Cytotoxicity of the isoflavone genistein in NIH 3T3 cells. Cell Biol. Int..

[20] Bose A., Baral R. (2007). Natural killer cell mediated cytotoxicity of tumor cells initiated by neem leaf preparation is associated with CD40–CD40L–mediated endogenous production of interleukin-12. Human Immunol..

[21] Ling L.T., Yap S.A., Radhakrishnan A.K., Subramaniam T., Cheng H.M., Palanisamy U.D. (2009). Standardised *Mangifera indica* extract is an ideal antioxidant. Food Chem..

[22] Miliauskas G., Venskutonis P.R., Beek T.A.V. (2005). Screening of radical scavenging activity of some medicinal and aromatic plant extracts. Food Chem..

[23] EPA (1996a). Method 3052 Microwave Assisted Acid Digestion of Siliceous and Organically Based Matrices. Test Methods for Evaluating Solid Waste, Physical/Chemical Methods.

[24] EPA (1996). Method 6010B Inductively Coupled Plasma-Atomic Emission Spectrometry. Test Methods for Evaluating Solid Waste, Physical/Chemical Methods.

[25] EPA (1991). Method 245.6. Determination of Mercury in Tissues by Cold Vapor Atomic Absorption Spectrometry, Revision 2.3..

[26] Palanisamy U., Cheng H.M., Masilamani T., Subramaniam T., Ling L.T., Radakrishnan A.K. (2008). Rind of the rambutan, *Nephelium lappaceum*, a potential source of natural antioxidants. Food Chem..

[27] Heo S.J., Hwang J.Y., Choi J.I., Han J.S., Kim H.J., Jeon Y.J. (2009). Diphlorethohydroxycarmalol isolated from *Ishige okamurae*, a brown algae a potent postprandial hyperglycemia in diabetic mice. Eur. J. Pharmacol..

[28] Da Silva Pinto M., Kwon Y.I., Apostolidis E., Lajolo F.M., Genovese M.I., Shetty K. (2009). Potential of Gingko biloba L. leaves in the management of hyperglycemia and hypertension using in vitro models. Bioresour. Technol..

